# Monitoring Health Inequalities in 12 European Countries: Lessons Learned from the Joint Action Health Equity Europe

**DOI:** 10.3390/ijerph19137663

**Published:** 2022-06-23

**Authors:** Pi Högberg, Göran Henriksson, Carme Borrell, Marius Ciutan, Giuseppe Costa, Irene Georgiou, Rafal Halik, Jens Hoebel, Katri Kilpeläinen, Theopisti Kyprianou, Tina Lesnik, Indre Petrauskaite, Annemarie Ruijsbroek, Silvia Gabriela Scintee, Milena Vasic, Gabriella Olsson

**Affiliations:** 1 Unit for Public Health Reporting and Evaluation, Public Health Agency of Sweden, 17182 Solna, Sweden; gabriella.olsson@criminology.su.se; 2 Region Västra Götaland, Regionens Hus, 40544 Göteborg, Sweden; goran.henriksson54@gmail.com; 3 Public Health Agency of Barcelona, Place Lesseps 1, 08023 Barcelona, Spain; cborrell@aspb.cat; 4 CIBER of Epidemiology and Public Health, Av. Monforte de Lemos 3-5, 28029 Madrid, Spain; 5 Centre for Health Services Research and Evaluation, National School of Public Health Management and Professional Development, Vaselor Str. 31, 021253 Bucharest, Romania; mciutan@snspms.ro (M.C.); sscintee@snspms.ro (S.G.S.); 6 Epidemiology Unit ASL TO3, Region Piedmont, Via Sabaudia 164, 10095 Grugliasco, Italy; giuseppe.costa@unito.it; 7 Administration Unit, Ministry of Health, 2 Prodromou & Chilonos Str. 17, Nicosia 1448, Cyprus; igeorgiou@moh.gov.cy; 8 Department of Population Health Monitoring and Analysis, National Institute of Public Health NIH—National Research Institute, Chocimska 24, 00-791 Warsaw, Poland; rhalik@pzh.gov.pl; 9 Division of Social Determinants of Health, Robert Koch Institute, General-Pape-Str. 62-66, 12101 Berlin, Germany; j.hoebel@rki.de; 10 Health and Welfare Promotion Unit, Finnish Institute for Health and Welfare, Mannerheimintie 166, 00270 Helsinki, Finland; katri.kilpelainen@thl.fi; 11 Health Monitoring Unit, Ministry of Health, 2 Prodromou & Chilonos Str. 17, Nicosia 1448, Cyprus; tkyprianou@moh.gov.cy; 12 Analysis and Development of Health, National Institute of Public Health, Trubarjeva 2, 1000 Ljubljana, Slovenia; tina.lesnik@nijz.si; 13 Institute of Hygiene, Didžioji Str. 22, LT-01128 Vilnius, Lithuania; indrepetrausku@gmail.com; 14 Centre for Nutrition, Prevention and Health Services, National Institute for Public Health and the Environment, P.O. Box 1, 3720 BA Bilthoven, The Netherlands; annemarie.ruijsbroek@rivm.nl; 15 Institute of Public Health of Serbia, Dr Subotica 5, 11000 Belgrade, Serbia; milena_vasic@batut.org.rs; 16 Faculty of Dentistry Pancevo, Zarka Zrenjanina 179, 13000 Pancevo, Serbia

**Keywords:** health inequalities, monitoring, health information systems, policy

## Abstract

To raise awareness about health inequalities, a well-functioning health inequality monitoring system (HIMS) is crucial. Drawing on work conducted under the Joint Action Health Equity Europe, the aim of this paper is to illustrate the strengths and weaknesses in current health inequality monitoring based on lessons learned from 12 European countries and to discuss what can be done to strengthen their capacities. Fifty-five statements were used to collect information about the status of the capacities at different steps of the monitoring process. The results indicate that the preconditions for monitoring vary greatly between countries. The availability and quality of data are generally regarded as strong, as is the ability to disaggregate data by age and gender. Regarded as poorer is the ability to disaggregate data by socioeconomic factors, such as education and income, or by other measures of social position, such as ethnicity. Few countries have a proper health inequality monitoring strategy in place and, where in place, it is often regarded as poorly up to date with policymakers’ needs. These findings suggest that non-data-related issues might be overlooked aspects of health inequality monitoring. Structures for stakeholder involvement and communication that attracts attention from policymakers are examples of aspects that deserve more effort.

## 1. Introduction

Population health in Europe is not improving in a uniform way. Socioeconomic health inequalities have largely persisted over the last decade, with varying patterns of trends across European countries [1,2]. Successful actions against health inequalities rest on the ability to measure and understand the problem on one hand, and to follow up on the impact of actions on the other [3]. Hence, to come to terms with health inequalities, access to valid data and a well-functioning health inequality monitoring system (HIMS) is crucial. 

Health inequality monitoring is a process of repeatedly observing how health inequalities between subgroups within populations change over time, as well as how the distribution of the social determinants of health changes and how determinants and health interact [4]. Health inequality monitoring practices vary across countries in Europe [5]. In some countries, it is an integral part of the national monitoring system, whereas, in other countries, the monitoring of health inequalities has only just started [5,6]. It has been concluded that the conditions for monitoring population health are often poorest in the countries with the poorest health status [5,7]. Why the preconditions for monitoring population health vary across countries is unclear. More research has been called for to learn more about the reasons, but also to explore the extent to which weaker systems translate into less effective policies [8]. This might be particularly relevant in relation to the monitoring of health inequalities. 

Health inequalities evolve from systematic differences in living conditions, circumstances, and opportunities across population groups and geographical entities. Typically, the higher one’s position in the social hierarchy, the greater the resources and the opportunities to act in health-enhancing ways. The unequal distributions of resources, opportunities, and scopes for action coupled with positions in the social hierarchy operate in different areas of life, across life’s course, and on different aggregate levels. The model by Diderichsen, Evans, Whitehead, et al. [9] is often used to illustrate the complex processes by which social conditions are linked to health inequalities. In brief, the model illustrates how socioeconomic position is associated with systematic differences in living conditions and with differential vulnerability to such conditions. Hence, the model not only postulates that people in lower social positions tend to be more exposed to harsh living conditions, such as stressful work environments and less social support, but also that the effect of such exposures might be stronger in those groups (differential vulnerability). This might be because they have fewer resources in terms of knowledge, networks, time, and/or money to handle constraints to which they are exposed, but also because such constraints tend to cluster and interact. This makes people in lower social positions more exposed to many risk factors simultaneously. A monitoring system of health inequalities should ideally relate to this inherent complexity by reflecting inequalities in health outcomes as well as the processes that give rise to them across life’s course and at different levels of aggregation. 

In Europe, current efforts to monitor health inequalities are often focused on comparing national averages or proportions of an outcome across nations or other geographical entities. Although averages provide relevant information, they are not sufficient to inform health equity-directed policies [5]. In order to provide policy-relevant information, health inequality monitoring has to look beyond national averages and explore health and preconditions for health in subgroups of the population. Regardless of whether the failure to do so is due to ignorance or to technical issues, such as unavailability of data, it is still likely to be a weak point in the monitoring of initiatives to reduce health inequalities. Varying health policy priorities and capacities among countries are likely to impose challenges to harmonized health inequality monitoring within the EU. If policy goals are to be taken seriously and if gaps are to be closed within a generation [3], current national HIMSs have to first better capture health inequalities and the processes underlying them. To induce changes that strengthen the practice of monitoring health inequalities in Europe, a necessary first step is to make visible any limitations in current monitoring practices.

This study was part of the three-year project Joint Action Health Equity Europe (JAHEE; 2018–2021), financed under the Third Health Programme of the European Union, and specifically work package (WP) 5. In JAHEE WP5, public health institutions representing 12 European countries worked jointly with the ambition to advance health inequality monitoring in Europe by attracting attention to, and improving, monitoring practices or capacities. 

Drawing from the experiences gained from JAHEE WP5, the overall aim of this paper is to report on the country assessments that were performed in the project to illustrate current opportunities and limitations within European countries to monitor health inequalities. By discussing from a joint European view what can be done to challenge the identified limitations or barriers, this paper may contribute to more effective strategies addressing health inequities, and particularly the role of HIMS to inform such strategies. More specifically, the paper aims to:

1. Identify preconditions for monitoring health inequalities in the 12 European countries;
2. Pinpoint common weak areas in current European HIMS and discuss steps to overcome them.

## 2. Materials and Methods

The work in JAHEE WP5 followed a structured work process. First, the partners worked jointly to create a commonly agreed framework for health inequality monitoring, describing the central components of an “ideal” HIMS according to the literature [10]. Building on this framework and on the different steps of the monitoring process described by the World Health Organization WHO [11], a survey was constructed to assess the current HIMS in each country. The country assessments aimed to identify gaps and areas of improvement in the health inequality monitoring capacities of each country in relation to the agreed ideal HIMS. Countries then used the assessments as a tool to identify and take actions related to monitoring according to their specific needs and respective starting points. 

The country assessments were carried out during the spring of 2019. The institutions responsible for answering the survey were either themselves responsible for public health monitoring in the 12 countries (e.g., health ministries) or were mandated by the competent authorities. The competences of the institutions involved are described in Table 1. The participating experts were nominated by their institutions on the basis of their respective roles as national experts in public health monitoring and skills and experience in inequality monitoring. They were encouraged to team up with additional expertise required, within or between institutions, to gain the inter-institutional competence necessary for completing the questionnaire. All 12 countries responded.

Information was collected regarding the status and scope of the current health inequality monitoring, including data availability, data analysis, reporting, evaluation, and the infrastructure of the system, using six sections and a total of 55 statements (Table 2). In the first section—Defining the system—eight statements were used to assess to what extent a strategy for health inequality monitoring is available, implemented, and recognized in the partner country. In the second section, eighteen statements were used to assess the availability and quality of current data sources, and especially the extent to which individual-level data are accessible. In the next section—Analyses—the availability of disaggregated data was assessed, as were the extents to which measures are used and analyses are conducted to allow for the monitoring of both the social gradient and more vulnerable groups. The section contained ten statements. In the Dissemination and Communication section, five statements were used to assess the means of communication and to what extent communication strategies are in place, stakeholders have been identified, and regular reporting of health inequalities is undertaken. The extent to which the HIMS is regularly evaluated and adapted in order to remain up to date and to properly reflect needs was assessed by six statements in the Evaluation section. In the last section—Infrastructure of the System—eight statements were used to assess the extent to which adequate and sufficient support in terms of, for instance, funding, human resources, leadership, training, knowledge, and technical tools, is considered available. 

Partners were asked to respond to each statement using a six-point Likert scale ranging from 1 to 6, where 1 is “Strongly agree”, 2 is “Agree”, 3 is “Agree somewhat”, 4 is “Disagree somewhat”, 5 is “Disagree”, and 6 is “Strongly disagree”. As the statements describe the ideal situation and 1 represents the strongest agreement, a higher score means greater challenges. This was also visible to the survey respondents by the colors of the alternatives, from green for 1–2 (indicating an ideal or near-ideal situation), over yellow (3–4), to red (5–6). The rating was conducted by one expert or reflected the consensus of two or more experts from each country. 

## 3. Results

The results from the country assessments, with responses to each of the statements within each of the six sections, are summarized per section.

### 3.1. Define the System

**Figure 1:**
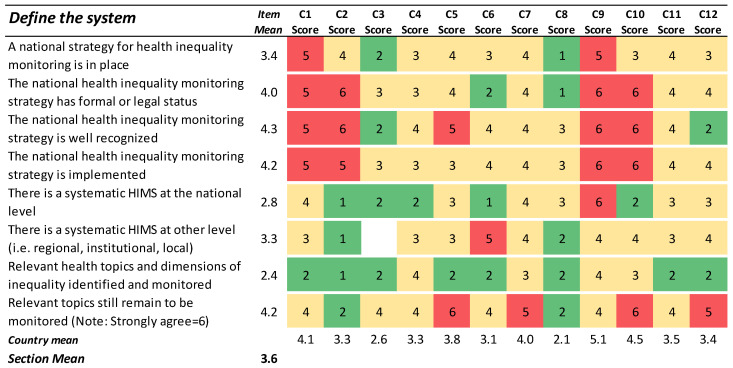
Results for the section ‘Define the system’. Green cells (scores 1–2) indicate agreement with the statement. Yellow cells (scores 3–4) indicate partial agreement/disagreement, and red cells (scores 5–6) indicate the most challenging preconditions for the ideal HIMS.

The results presented in Figure 1 show the partners’ responses in relation to statements related to structure and strategy in the country assessment. As indicated by the many red cells and the relatively high total section mean (score: 3.6), many partner countries disagreed with the statements in this section. The item responses indicate that many countries lack strategies for health inequality monitoring or, if strategies exist, that the implementation or recognition of the same is regarded as insufficient. 

On the other hand, regarding the question of whether there was a systematic HIMS at the national level in place, almost half of the countries (5 out of 12) concurred. All five national HIMS were also described by the respective experts as improving, meaning that they were undergoing fine-tuning or similar (*data not shown*). The difference between ratings of 1 or 2 on this statement was marginal, whereas a rating of 3 or higher clearly indicated that a systematic HIMS was not yet in place at the national level.

### 3.2. Data

**Figure 2:**
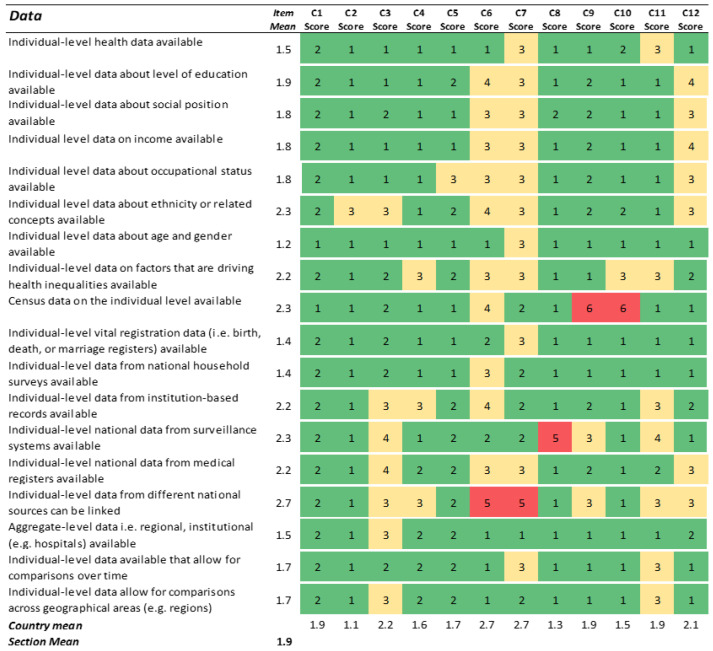
Results for the section ‘Data’. Green cells (scores 1–2) indicate agreement with the statement. Yellow cells (scores 3–4) indicate partial agreement/disagreement, and red cells (scores 5–6) indicate the most challenging preconditions for an ideal HIMS.

The availability of data was regarded as good in most countries, as shown by the many green cells and relatively low total section mean (1.9) in Figure 2. Overall, across sections, the data dimension thus seemed to be the least problematic area of the monitoring cycle, although there was considerable variation between countries. The partners generally reported rather good availability of individual-level data regarding health, social determinants of health, gender, and age, and relatively good availability of individual-level data on income, education, and occupation. Lower availability of individual-level data was reported for ethnicity or related concepts, such as time of residence, being foreign-born, and country of birth. Some countries show room for improvement in the availability and linkability of certain data. One example is country 7, where the given reason for scoring 5 on the linkability of individual data was legal restrictions, a situation that was also regarded as worsening.

### 3.3. Analyses

**Figure 3:**
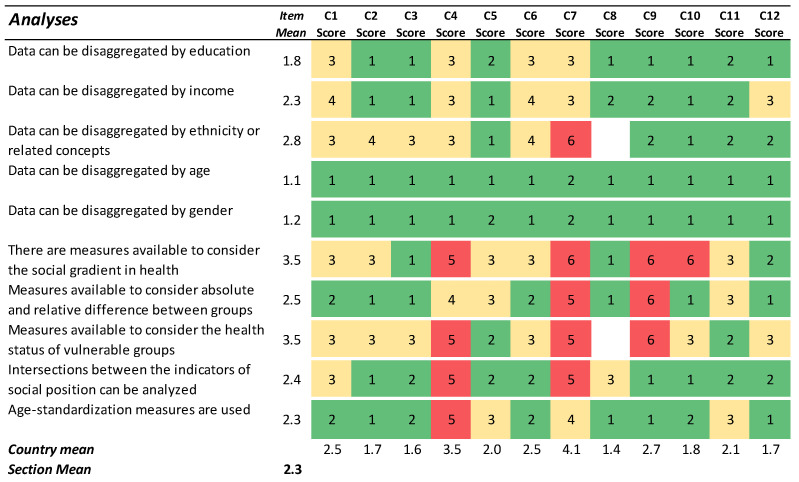
Results for the section ‘Analyses’. Green cells (scores 1–2) indicate agreement with the statement. Yellow cells (scores 3–4) indicate partial agreement/disagreement, and red cells (scores 5–6) indicate the most challenging preconditions for an ideal HIMS.

The responses in the Analyses section varied greatly across countries and between statements, especially with regard to the ability to stratify data (Figure 3). As suggested by the many green cells in relation to the statements about the ability to stratify data by gender or age, this ability was generally regarded as good. Regarding the ability to stratify data along a measure of social position, the results suggest that this ability was generally regarded as poorer, especially in relation to ethnicity or related concepts. The ability to assess the health status of vulnerable groups, such as the unemployed, the population at risk of poverty, and ethnic minority groups, was generally rated as poor.

Three out of the twelve countries used measures to assess the social gradient in health (slope index of inequality and/or relative index of inequality; *data not shown*), and hence provided a score of 1 or 2 to the sixth statement in Figure 3. However, two of those three countries added that they did not use those measures by routine (*data not shown*). Encouragingly, eight out of the twelve countries (even among those rating 5 or 6 for this item), described measurements of the social gradient in health as under development, prioritized, and/or improving (*data not shown*). Seven countries agreed with the statement that there are measures in place to calculate the absolute and relative differences between two contrasting groups. An additional country (C5) scored 3 on this item, but added information revealing that such measures are indeed in place. 

### 3.4. Dissemination and Communication

**Figure 4:**
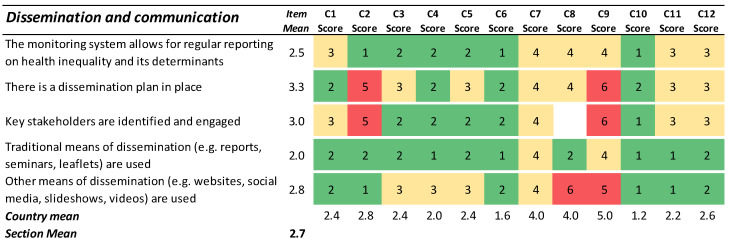
Results for the section ‘Dissemination and Communication’. Green cells (scores 1–2) indicate agreement with the statement. Yellow cells (scores 3–4) indicate partial agreement/disagreement, and red cells (scores 5–6) indicate the most challenging preconditions for an ideal HIMS.

The preconditions for dissemination and communication were generally regarded as good in two countries (C6 and C10), whereas the remaining ten countries showed more room for improvement (Figure 4). A closer look at the item responses suggested that, in most countries, the monitoring system is formed in a way that allows the dissemination of results on health inequality on a regular basis. The item mean of 2.0 for the use of traditional means of dissemination indicates that countries are reporting more or less regularly on the outcomes of their existing health inequality monitoring. However, the extent to which there is a dissemination plan in place, stakeholders are engaged with, and non-traditional means of communication are used, varied largely between countries.

### 3.5. Evaluation

**Figure 5:**
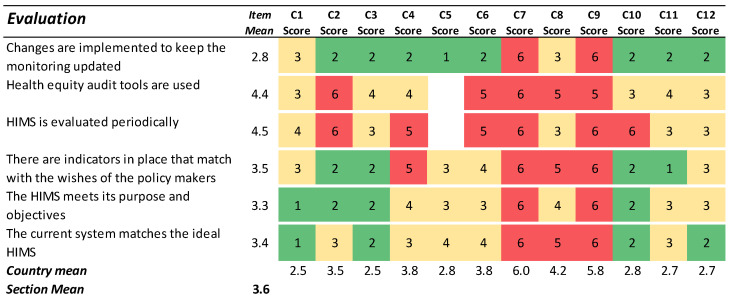
Results for the section ‘Evaluation’. Green cells (scores 1–2) indicate agreement with the statement. Yellow cells (scores 3–4) indicate partial agreement/disagreement, and red cells (scores 5–6) indicate the most challenging preconditions for an ideal HIMS.

The section Evaluation investigates the knowledge about whether the HIMS is useful to stakeholders and the extent to which the results from health inequality analyses are implemented in policymaking (Figure 5). The overall level of agreement with the statements in this section was poor, as indicated by the many red cells. Specifically, the responses suggested that the output from HIMS was rarely evaluated systematically to ensure reliability, validity, or adherence to new regulations and new technology. Nor do there seem to be routines in place to ensure the adequacy of the monitoring practice for policymakers. 

### 3.6. Infrastructure of the System

**Figure 6:**
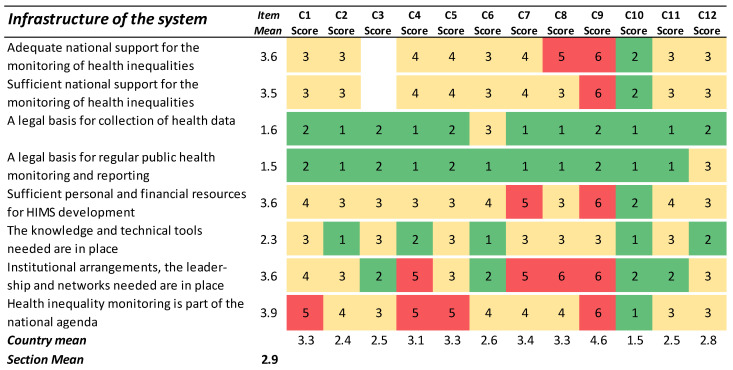
Results for the section ‘Infrastructure of the system’. Green cells (scores 1–2) indicate agreement with the statement. Yellow cells (scores 3–4) indicate partial agreement/disagreement, and red cells (scores 5–6) indicate the most challenging preconditions for an ideal HIMS.

The results in this section capture the extent to which the resources and capacities needed for an appropriate HIMS are available (Figure 6). The responses here suggest that, while the legal basis for regularly collecting data and reporting on results was less problematic, the preconditions in terms of personal and financial resources, capacity building, and institutional arrangements were insufficient or lacking.

## 4. Discussion

In several places in Europe, steps are being taken toward establishing a more optimal HIMS. In line with what has been concluded elsewhere [5], this study of 12 European countries has shown that many of the challenges that impede the progress toward better health inequality monitoring are often common across countries, and there is scope for improvement in all countries. Summing up the overall results of the six sections of the country assessment, generally strong areas in existing systems to monitor health inequalities seem to be related to data availability and quality, and to some extent, data analysis. However, few countries have a proper health inequality monitoring strategy in place that is well recognized and implemented. Current health inequality monitoring practices are often regarded as moderately or poorly evaluated and are not ensured to be up to date with the needs of policymakers or with technical innovations. These results also have implications for the possibility to create an international HIMS, harmonized and agreed at the European level, which could be used to discuss health inequity in Europe more effectively.

Despite its simple format, the study provided important insights. Firstly, improvements are still needed in terms of the ability of countries to systematically use disaggregated data by relevant social stratifiers in the monitoring of health inequalities. This will be further discussed below. Secondly, as weaker areas were related to the perceived appropriateness of the current HIMS for policymakers, we found reason to believe that the development of a sustainable HIMS would benefit from more strategic considerations, communication, and evaluation. The modest efforts in communication that showed to be concurrent may well be related to the perceived inappropriateness of the current HIMS for policymakers, or even ignorance from policymakers to health inequalities. A third important insight is the large variation in preconditions and the differing contexts in which information systems for health inequalities are to be improved and better aligned. 

### 4.1. Strategy-Related Challenges

Many countries lacked well-accepted and implemented strategies for health inequality monitoring. Ideally, such strategies would define the objectives of health inequality monitoring, identify which health topics and social determinants of health should be included, and visualize how the outputs are to be implemented in policymaking [4,12]. If the strategy does not have legal or formal status, or is not tied to some form of political commitment, it will be difficult to organize a systematic and sustainable HIMS where the results will be used as a basis for decisions on actions to improve public health. The largest strategy-related challenge was shown for the statement ‘The national health inequality monitoring strategy is well recognized’. Regardless of whether there is a strategy in place or not, this suggests that communication is an often-neglected aspect that could, if improved, lead to a stronger political commitment that is needed to build or reinforce a HIMS. 

### 4.2. Analysis- and Infrastructure-Related Challenges

When it comes to understanding how socioeconomic position, ethnicity, or similar concepts are linked to health outcomes, the common poor availability of disaggregate data on health and drivers of health inequalities by socioeconomic or migrant status constitutes a major obstacle to improvements. The country assessments indicated that, although several countries have access to data on education, occupation, income, and migration status (Figure 2), fewer countries reported the ability to disaggregate data by such stratifiers (Figure 3). Given that the latter information stems from a more specific and concrete question, this probably better reflects the real situation when it comes to developing a more ideal national HIMS. In any case, if acquiring data for stratification on, for example, education is a real challenge, other approaches might be useful, such as using information from small geographical areas as a proxy for social position [13,14]. Indeed, one of the twelve studied countries reported using an index of spatial deprivation to monitor health inequality.

Legal restrictions were put forward by some countries as obstructing the analyses of data linked to the socioeconomic or ethnic background of individuals. Indeed, the General Data Protection Regulation (GDPR) puts restrictions on the processing of individual data in order to protect the privacy and security of individuals. However, the GDPR also provides some exceptions from such restrictions [15]. There are still few legal precedents on this topic, and thus it is too early to judge what consequences these restrictions will have on the ability to monitor health inequalities. In any case, it is important that the public health community raises awareness of the delicate balance between protecting personal integrity on one hand and the need to improve our ability to monitor health inequalities on the other. 

Another central challenge related to analysis is to decide by which measures health inequalities should optimally be assessed. To better understand the degree of health inequality, it is important to avoid simplifications, such as crude geographical comparisons or proportions of health among disadvantaged groups, because these only consider one side of the social gradient. However, our study showed that complex inequality measures, which capture the social gradient of a particular health indicator, are rarely used in current health inequality monitoring in the studied countries. Another challenge is that intersectionality provides a complexity that is rarely considered. The literature usually suggests that health inequalities should be assessed using several different measures, as there is no single measure that covers all relevant aspects of inequalities, and different socioeconomic indicators can tap into different causal mechanisms [4,16,17]. The example from Scotland may provide inspiration for this topic [18]. In the Scottish HIMS, systematically used measures are the absolute range, the relative index of inequality (RII), and a measure of scale (by using an income–employment index). With these, three questions are answered as a snapshot and as changes over time—the gap in health inequality, the steepness of the health gradient, and the underlying scale of the problem. In any case, the answer to the question of which measures of health inequality to apply will, by necessity, be a compromise between justified complexity and comprehensibility by laymen. 

Financial challenges related to infrastructure were also put forward in the country assessments. Some countries rated the preconditions as insufficient or lacking in terms of personal and financial resources and institutional arrangements. However, it remains to be shown whether an optimal HIMS is more expensive than a suboptimal HIMS and how to best organize institutional capacities. 

### 4.3. Communication- and Evaluation-Related Challenges

Our results indicate that there is significant room for improvement concerning the appropriateness of existing HIMS for policymakers and the involvement of stakeholders in strategic dialogues on monitoring. Non-data-related issues, e.g., organization, funding, stakeholder involvement, and political commitment, might thus be overlooked aspects of monitoring health inequality. Such aspects become interesting to investigate when knowing that only a few scientifically sound policy recommendations to reduce health inequalities have been transformed into actual policy changes, as noticed by scholars [19,20]. There are probably several reasons for this. For example, while the concept of The Social Determinants of Health has provided a useful framework for the public health community, it has been difficult to “translate” the framework to other sectors, professions, policymakers, and academic disciplines [21]. Another reason may be related to the inherently political nature of inequalities and the question of to what extent the scientific community should keep a “neutral” stance toward politics [22]. Both reasons are strong arguments for dialogue between policymakers, professionals, and researchers on how to align health inequality monitoring with policy objectives, provided that there is a sincere political will to employ evidence-based interventions. However, this kind of dialogue does not yet seem to be in place, as our study and others [23] have concluded. A third reason contributing to the modest communication and evaluation of monitoring practices could be the fact that some experts did not regard the country as having any monitoring of health inequalities in place. As a consequence, communication and evaluation will, of course, also be lacking. In whatever case, communication and dialogue with stakeholders about needs is likely to be a key next step.

Therefore, countries may need to form fora for discussion between policymakers, professionals, and other interested parties of determining what the health policy objectives are, which indicators are appropriate to measure developments toward the goals, which measures to use, and how to disseminate results. For optimal use in policy, the results from monitoring, i.e., the numbers, need to be accompanied by an interpretation of how the numbers link to amenable mechanisms, drawing from current knowledge. Such knowledge may be visualized by interrelations of indicators through the communication of causal networks, rather than causal chains [24].

### 4.4. Strengths and Limitations

The work presented here is part of a large EU-funded joint action, sharing the strengths associated with such projects. Quality checkpoints during the planning, execution, and evaluation of the project were ensured through the organizational structure, which included a scientific advisory board, a policy advisory board, and internal as well as external evaluators. Another strength associated with large EU joint actions, such as the JAHEE, is the representation of experts from a large number of countries, ensuring a certain extent of representativeness of the EU’s situation. The results presented here should, however, not be interpreted as generalizable to the EU, since participating countries were not randomly selected, but rather applied to join the JAHEE. 

A limitation of the method of country assessments using expert opinion is that it is inherently influenced by subjective evaluation criteria as well as the selection of experts involved in the survey response. As a result, there may be a risk of reporting bias. However, the national experts in this project had experience working together in the JAHEE with a common objective to improve abilities to monitor health inequalities in the national context. The survey answers for each country were provided by collaborative teams comprised of officials and researchers under the coordination of an appointed contact person. The survey instructions also emphasized that, in cases where the respondents did not have access to the requested information, they were to describe why. With all these factors taken into consideration, we believe that the obtained results reflect neither a gross overestimation nor an underestimation of the reported capabilities and resources.

The answers were independently given per country and not calibrated against the answers of other countries. Hence, our results should not be used for comparing country scores against each other, despite being the best possible assessments of the within-country situation. We deliberately looked for joint patterns, rather than focusing on individual countries or country comparisons, in order to provide general guidance for the development of robust and policy-relevant health inequality monitoring.

The COVID-19 pandemic struck during the project period, which limited the project to some extent, but also acted as a catalyst as the pandemic served as a wake-up call for all of Europe to act on health inequities. By building upon the results of the country assessments, partner countries have been able to pilot or fine-tune their monitoring to provide evidence on the strikingly different health effects of the pandemic on different socioeconomic groups. 

## 5. Conclusions

The preconditions, opportunities, and challenges to developing sustainable and policy-relevant national HIMS show large variations across Europe. We conclude that data access is the least challenging area, although there is still a need for improvement, particularly concerning the disaggregation of health data by social stratifiers and linkage of data. A country that wishes to develop a HIMS or improve its existing HIMS may, therefore, consider whether the next step should be about expanding indicators or about strengthening infrastructure and capacity, the latter including communicating along the developmental and implementation stages to ensure the active involvement of policymakers and other stakeholders in the process.

## Figures and Tables

**Table 1:** Affiliations of the experts participating in the country assessments and description of the institutional competence for this task.

Partner Country	Experts’ Affiliations	Institutional Competence
Cyprus	Ministry of Health Cyprus—Health Monitoring Unit	Collects and compiles national data on health
	Cyprus Statistical Service	Collects and compiles data on population, health, social service, living conditions and social protection, poverty, and social exclusion
Finland	National Institute for Health and Welfare (THL)	Independent expert agency under the Ministry of Social Affairs and Health. Studies, monitors, and develops measures to promote the well-being and health of the population in Finland, including health equity
Germany	Robert Koch Institute (RKI), Department of Epidemiology and Health Monitoring	Administers the German national health monitoring system on behalf of the German Federal Ministry of Health
Italy	The Italian Institute of Statistics (Istat)	Census, mortality, surveys, and health indicator data
	The network of central institutions: Ministry of Health, Dept. of Health Information Systems (a), Istat (b), INPS (c), INAIL (d), and INAPP (e)	(a) Health care data, (b) mortality and survey data, (c) employment and retirement data, (d) work injury and occupational diseases data, and (e) public policy evaluation
	Piedmont Regional Health Authority	Regional health observatory, needs/risk analysis, assessment of potential solutions, and monitoring and evaluation of processes and outcomes of interventions and policies
	Regional network of experts on health inequalities indicators: units of Piedmont, Lazio, Emilia Romagna, Toscana, INAPP, and AgeNaS	The main composite indicators of social deprivation and income at census tract and municipality levels
Lithuania	Institute of Hygiene (HI), Division of Biostatistical Analysis	Institution responsible for monitoring and reporting on health data including, but not limited to, mortality, morbidity, and health inequalities
The Netherlands	National Institute for Public Health and the Environment (RIVM), Centre for Nutrition, Prevention and Health Services	Research on and monitoring of health inequalities in close cooperation with universities and Statistics Netherlands
Poland	National Institute of Public Health NIH—National Research Institute	Main governmental institute in charge of monitoring public health and health inequalities, also responsible for disseminating knowledge to policymakers and other stakeholders in the area of health about the health situation of Polish society and best practices in public health
Romania	National School of Public Health, Management and Professional Development Bucharest	Adviser to Ministry of Health policies
	National Institute for Mother and Child Health (NIMCH)	Coordinator of the national program for mother and child health, collecting data related to this area
Serbia	Institute of Public Health of Serbia “Dr Milan Jovanovic Batut”	National expert institution for Public Health, including data collection and maintenance, analysis, planning, and organization
Slovenia	Center of Health Analysis and Development of Health	Central national institution in public health, provides expert support to governmental decisions
Spain	Public Health Agency of Barcelona (ASPB). The assessment made by ASPB was shared afterward with professionals working in the Ministry of Health of Spain.	Monitors and reports on population health status, health determinants, and health inequalities using indicators at the area level, mainly in Barcelona. Develops and implements public health policies and interventions to reduce health inequalities
Sweden	Public Health Agency of Sweden	Independent national governmental authority assigned to collect data, monitor and report on health, health determinants, health threats, and health inequalities

**Table 2:** Overview of survey sections, what was assessed in each section, and the number of items (statements) per section.

Section	What Was Assessed	Number of Items
Defining the system	To what extent a strategy for monitoring health inequality is available, implemented, and recognized	8
Data	The availability and quality of current data sources and the extent to which individual-level data are accessible	18
Analyses	The availability of disaggregated data and the extent to which measures and analyses are used to allow for monitoring both the social gradient in health and vulnerable groups	10
Dissemination and communication	To what extent a communication strategy is tied to health inequality monitoring, with identified stakeholders and regular reporting	5
Evaluation	To what extent the HIMS is regularly evaluated and adapted in order to remain up to date and to properly reflect needs	6
Infrastructure	Availability of adequate and sufficient support for health inequality monitoring, e.g., funding, human resources, leadership, training, knowledge, and technical tools	8

## Data Availability

Publicly available datasets were analyzed in this study. The data are available on request from the Public Health Agency of Sweden, by contacting the Registrar and stating diary number 03265-2019.
